# The molecular mechanism of nitric oxide in memory consolidation and its role in the pathogenesis of memory-related disorders

**DOI:** 10.1007/s10048-025-00803-0

**Published:** 2025-01-24

**Authors:** Zainab I. Bahdar, Ejlal Abu-El-Rub, Rawan Almazari, Ayman Alzu’bi, Raed M. Al-Zoubi

**Affiliations:** 1 Department of Basic Medical Sciences, Faculty of Medicine, Yarmouk University, Irbid, 211-63 Jordan; 2 Department of Surgery, Surgical Research Section, Hamad Medical Corporation, Doha, Qatar; 3 Department of Biomedical Sciences, QU-Health, College of Health Sciences, Qatar University, Doha, 2713 Qatar; 4 Department of Chemistry, Jordan University of Science and Technology, P.O.Box 3030, Irbid, 22110 Jordan

**Keywords:** Nitric oxide, Short-term memory, Long-term memory, Molecular mechanism, Pathogenesis

## Abstract

Memory is a dynamic process of encoding, storing, and retrieving information. It includes sensory, short-term, and long-term memory, each with unique characteristics. Nitric oxide (NO) is a biological messenger synthesized on demand by neuronal nitric oxide synthase (nNOS) through a biochemical process initiated by glutamate binding to NMDA receptors, causing membrane depolarization and calcium influx. NO is known to regulate many signaling pathways including those related to memory consolidation. To throw light on the precise molecular mechanism of nitric oxide (NO) in memory consolidation and the possibility of targeting NO pathways as a therapeutic approach to scale down cognitive impairments. We conducted a search of the PubMed MEDLINE database, maintained by the US National Library of Medicine. The search strategy utilized Medical Subject Headings (MeSH) terms, including “nitric oxide and memory,” “nitric oxide synthesis in the brain,” “nitric oxide and Alzheimer’s,” “nitric oxide and Parkinson’s,” and “nitric oxide, neurodegenerative disorders, and psychiatric disorders.” Additionally, relevant keywords such as “nitric oxide,” “memory,” and “cognitive disorders” were employed. We included the most recent preclinical and clinical studies pertinent to the review topic and limited the selection to articles published in English. NO exerts its role in memory consolidation by diffusing between neurons to promote synaptic plasticity, especially long-term potentiation (LTP). It acts as a retrograde messenger, neurotransmitter release modulator, and synaptic protein modifier. The dysregulation of NO balance in the brain can contribute to the pathogenesis of various neurodegenerative diseases, particularly Alzheimer’s, Parkinson’s, and psychiatric disorders. The disturbance in NO signaling is strongly correlated with synaptic signaling dysfunction and oxidative stress. NO plays a fundamental role in memory consolidation, and its dysregulation contributes to cognitive impairment—a hallmark of numerous neurodegenerative and psychiatric disorders. Future research should aim to deepen our understanding of the mechanisms underlying NO’s involvement in memory consolidation and to explore therapeutic strategies targeting the NO pathway to mitigate cognitive decline in affected individuals.

## Introduction

**Fig. 1:**
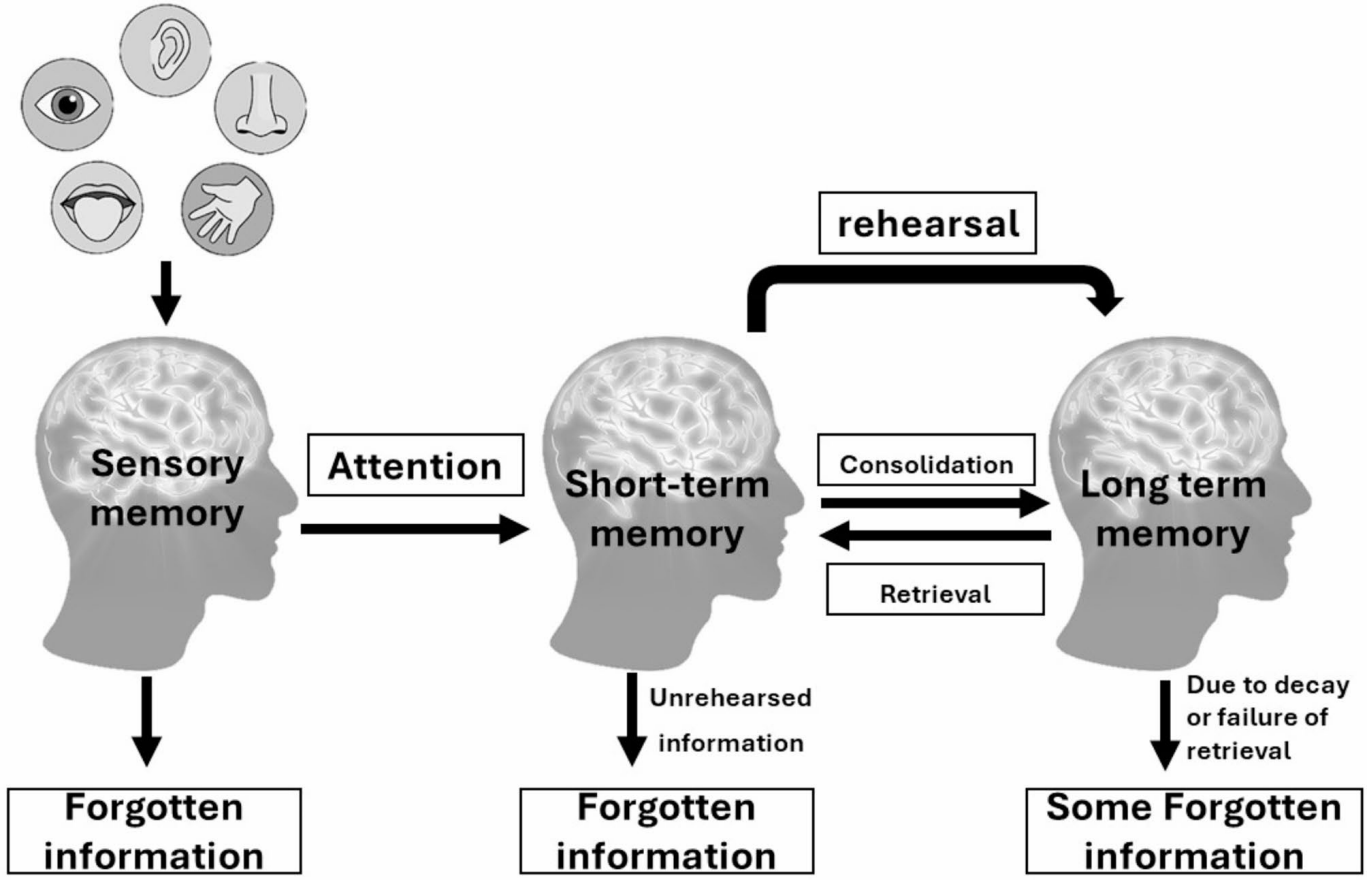
The Atkinson–Shiffrin memory model. This model describes the three stages of memory formation: sensory memory, short-term memory, and long-term memory. It also highlights the importance of rehearsal to pass through these stages and form a long-term, life-long memory

Memory is a dynamic chemical process between neurons [1, 2]. Many scientists have concurred to define memory as the cognitive ability that allows the human high brain centers to encode, store, and retrieve information when needed; it is the perpetuation of information for future influencing act [3], like adapting, learning, solving problems, and making decisions. According to the Atkinson–Shiffrin memory model (Fig. 1) that was proposed in 1968, information that enters our brain and forms a memory passes through three co-depended stages; sensory memory, short-term memory (STM), and long-term memory (LTM) [4].

Sensory memory refers to the memory formed for sensorial details of a life situation, this includes any detail that can be obtained by our five senses: visual, auditorial, tactile, olfactory, and gustatorial [5]. It allows us to recognize a familiar scene, voice, or the face of someone we know. It can accurately receive this information but ultimately fades away [6]. Although this memory has a great capacity, these sensorial details last only for milliseconds [6] and are mostly outside our conscious awareness as these memories are captured through the auditorium and visual pathways, and then sent to be relayed on the thalamus through the sensory cortices [7, 8].

Short-term memory (STM) refers to the retention of details and information in conscious awareness for only a few seconds, and it is limited in capacity. In fact, an erudite theory developed by Miller in the 1950s states that short-term memory holds seven units of information, plus or minus two items, although new research suggested that it is closer to four items, and other research proposed that it is reliant on the individual’s processing speed [9]. The information obtained in short-term memory will eventually leave the processing system leading to either an evanescent response or being stored in the long-term memory [9]. The Atkinson and Shiffrin model suggested that unless we rehearse the obtained information verbally, the information can stay in the STM for 15–30 s before we start losing it [6, 10].

LTM refers to the memory that is held for a longer period or considered everlasting It has an enormous capacity that store complicated knowledge structured into cognitive dynamic schemas, which can be alternatively defined as distinct pieces of obtained knowledge that organize and categorize the data [6]. The LTM is primarily the function of the hippocampus and is stored throughout the cortex [11]. Long-term memories can be classified into two categories: procedural (or implicit) and declarative (or explicit). Procedural memory is the memory that forms by implementing and repeating exposure to a series of motor outputs [11]. Declarative memory is the process of forming intentional memories by the direct recollection of event content and can be further sub-classified as episodic memory about personal experiences and their related details and semantic memory about factual knowledge [12].

NO is a small and highly diffusible neurotransmitter that acts as a biological messenger. It possesses many unique characteristics and participates in many cellular signaling pathways. It is not synthesized after membrane depolarization and is not stored in synaptic vesicles like many neurotransmitters, nevertheless, it is made and released upon cellular needs. In addition to that, NO doesn’t perform its function by binding to receptors on the targeted membrane, but by diffusing between the neurons and acting directly on the cell’s intracellular components [13]. This potent molecule plays an essential role in many physiological processes within the body cells, particularly in the nervous system as a neurotransmitter and neuromodulator. In the central nervous system (CNS), nitric oxide has different roles, including the regulation of synaptic plasticity, the sleep-wake cycle, and hormone secretion [14]. It largely plays a vital role in enhancing the functionality of the hypothalamus pertaining to the learning process and memory formation [13, 15].

To obtain the desired level of NO, a series of biochemical steps must be meticulously followed. NO synthesis starts with Glutamate binding to the NMDA receptor which will eventually cause a membrane depolarization and the influx of calcium. Calcium will bind to calmodulin forming the co-factor complex; the calcium-calmodulin cofactor, which activates the nitric oxide synthase (NOS). NOS then acts on the primary substrate L-arginine, which is taken up by the cationic amino acid transporter (CAT-1). L-arginine will then be hydroxylated first into N-hydroxy-L-arginine, and then oxidized into two molecules: L-citrulline and nitric oxide [13].

The recognition of nitric oxide’s role in memory consolidation is vital, thus it is considered the mainspring in understanding the physiology of cognition and an important target for current and future therapeutic applications. Despite the continuous advancement in comprehending many aspects in the field of neuroscience, there are still gaps that need to be filled about the precise mechanism by which NO influences memory consolidation, or what are the conditions by which NO exerts its effect. Based on that our minireview aims to emphasize the intriguing implication of nitric oxide in memory formation and the impact of NO dysregulation in memory-related diseases. This review looks further at how to use nitric oxide as a target for treating or reducing the disease severity in memory-related disorders such as Alzheimer’s disease, and other forms of cognitive impairment related to psychiatric disorders.

## Nitric oxide formation and its mechanism in memory consolidation

### Nitric oxide formation

**Fig. 2:**
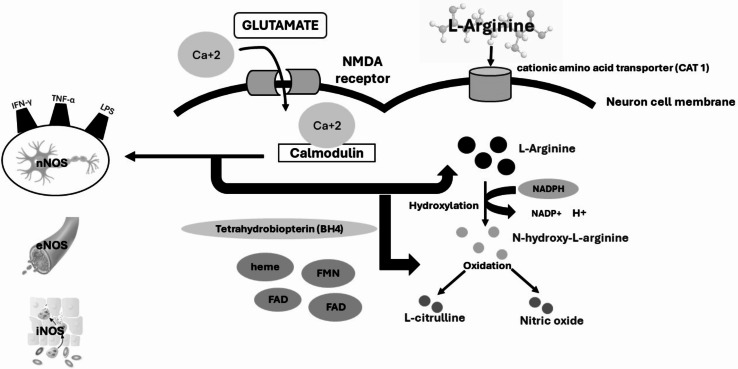
The biochemical steps for NO synthesis and the important molecular factors required for its production

Nitric oxide has multiple regulatory functions in our central nervous system, ranging from vasodilation to neurotransmission, and immune defense mechanisms. Nitric oxide synthesis in our central nervous system is primarily catalyzed by the nitric oxide synthase (NOS). The NOS has three isoforms: endothelial NOS (eNOS), neuron NOS (nNOS), and inflammatory NOS (iNOS). Despite that both eNOS and iNOS are present in the CNS, nNOS is the major enzyme responsible for neuronal NO production [16]. nNOS gene is regulated at the transcriptional level. Sp1 and CREB both regulate the initiation of gene transcription in response to the activation of neurons. This transcription will lead to the translation of mRNA into the nNOS in the cytoplasm, where it will undergo posttranslational modification. nNOS will interact with the calcium calmodulin complex, which was previously formed when glutamate is bound to the NMDA receptor, allowing depolarization of the membrane, and causing an influx of calcium. To get nitric oxide as a by-product, we start with the substrate L-arginine that enters the cytoplasm through the cationic amino acid transporter (CAT1) transporter. nNOS will interact with L-arginine causing hydroxylation into N-hydroxy-L-arginine and then oxidized with the aid of the co-factor tetrahydrobiopterin (BH4) that couples to the nNOS enzyme to get two products: L-citrulline and nitric oxide. Thus, the absence of the substrate or BH4 does not result in the production of nitric oxide [17]. This absence might be caused by conditions that increase oxidative stress, such as UV radiation and ionizing radiation, resulting in NOS uncoupling and the generation of highly oxidative free radicals [18]. Other co-factors include NADPH, heme, Flavin Adenine Dinucleotide (FAD), and Flavin Mononucleotide (FMN) all involved in transferring electrons within the enzyme complex, thus facilitating the conversion of L-arginine to nitric oxide (NO) and L-citrulline. The nitric oxide will then diffuse to the postsynaptic cleft to exert its multiple effects on nearby neurons [19]. The biochemical steps of Nitric oxide synthesis and the factors required to speed up nitric oxide production have been illustrated in Fig. 2.

### Nitric oxide mechanism in memory consolidation

Memory consolidation is defined as the complex process in which the temporary volatile memory is changed into a more stable, long-lasting form [20]. Nitric oxide plays a pivotal role in memory consolidation, via its involvement in synaptic plasticity, a fundamental process in which the connection between the neurons can ‎strengthen or weaken over time according to their activity, which is crucial for encoding and tracing ‎storage of the type of memory mediated by the brain area in which is observed [21]. Nitric oxide participates in synaptic plasticity through enhancing long-term potentiation (LTP) in the hippocampus and cerebral cortex [22].

Nitric oxide affects synaptic plasticity through different pathways to facilitate this transmission of memories into a more stable, permeant form. it functions as a retrograde messenger, modulating neurotransmitter release, regulating the dynamics of synaptic proteins, and impacting gene expression and protein synthesis, NO ensures that neural circuits are optimally configured for the long-term retention of information. This multi-pathway clears the air about how nitric oxide is pivotal in cognitive functions and its potential for future therapeutic targeting strategies in treating memory-related disorders [23].

### Nitric oxide as a retrograde messenger

Nitric oxide serves as a retrograde ‎messenger after postsynaptic NMDA (N-methyl-D-aspartate) receptor activation in hippocampal long-term potentiation ‎‎(LTP) [22]. It all starts when the NMDA receptors on the postsynaptic are activated, leading to the influx of calcium ions (Ca + 2) [24]. This influx activates neuronal NOS leading to synthesizing the final product nitric NO. NO then diffuses rapidly to the presynaptic cleft where soluble guanylate cyclase (sGC) is activated. sGC catalyzes the conversion of GTP to cyclic GMP (cGMP), where the increase in cGMP causes the activation of protein kinase G [25]. Protein kinase G (PKG) in turn phosphorylates multiple proteins involved in NO release, such as glutamate, thus enhancing the synaptic strength and promoting LTP. The abovementioned complicated signaling cascade emphasizes the importance of NO in regulating synaptic plasticity and memory consolidation [25].

### Nitric oxide modulation of S-nitrosylation of proteins

Nitric oxide also plays a role in modulating neurotransmitter release through various pathways, one of which involves modulating the release through the S-nitrosylation of proteins. S-nitrosylation is a post-translational modification of proteins in which the nitric oxide is covalently bound to the free sulfhydryl group of specific cysteine residues of the targeted protein, resulting in the formation of S–S-nitrosothiol (SNOI) [26]. For instance, S-nitrosylation can impact protein-protein interactions, either promoting or inhibiting the binding of proteins to their cognate partners. The S-nitrosylation of NMDA (N-methyl-D-aspartate) receptors were found to affect the calcium influx, therefore having an impact on the learning and memory process [27]. The enhancement of the NMDA receptor’s activity via S-nitrosylation will cause an increase in calcium influx, promoting synaptic plasticity [28]. The S-nitrosylation has a dual role of being neuroprotective and neurotoxic. It can protect the nervous system by inhibiting harmful processes such as excessive NMDA receptor activity, which can lead to excitotoxicity and neuronal damage [2]. Meanwhile, an abnormal concentration of S-nitrosylation can interfere with mitochondrial function leading to impaired energy production, synaptic damage, and increased oxidative stress, and collectively can cause neurotoxic effects which are implicated in multiple neurological disorders [2, 29]. Nitric oxide-mediated S-nitrosylation enhances NMDA receptor stimulation, thereby promoting calcium influx and synaptic plasticity, both of which are required for memory retention and retrieval [30]. A study by Forrester et al. (2009) described the biotin-switch technique (BST), a commonly ‎used assay for detecting S-nitrosylated proteins in complicated biological systems. This approach ‎has proved useful in understanding the conducted role of nitric oxide in modifying protein S-nitrosylation, which ‎influences a variety of cellular processes such as neurotransmitter release and synaptic plasticity. ‎BST has shed light on the ‎molecular mechanisms governing memory preservation and retrieval by allowing the identification and quantification of S-nitrosylated proteins‎ [31]. Building upon the role of NO in modulating synaptic activity through S-nitrosylation, it is also known to influence the activity of calcium/calmodulin-dependent protein kinase II (CaMKII), a major enzyme involved in synaptic plasticity and memory processes. One study found that NO suppresses CaMKII activity via S-nitrosylation, affecting synaptic function and memory functions [32]. This study found that reducing agents can reverse NO-mediated inhibition of CaMKII, showing that S-nitrosylation is reversible and NO plays a dynamic function in controlling synaptic plasticity.

### Nitric oxide modulation of Calcium/Calmodulin-Dependent kinases (CaMKs)

CaMK (calcium calmodulin-dependent kinase) is a major holoenzyme of the postsynaptic density that applies its function through phosphorylating key receptors such as the NMDA and AMPAR [33]. Upon calcium influx, CaMK undergoes autophosphorylation, resulting in prolonged Ca2+/CaM-independent autonomous kinase activity, which brings out the prolonged enhancement of LTP [34]. Notably, NO-cGMP signaling pathways intersect with CaMK pathways, further influencing their activity and contributing to the highest, intricate levels of neuronal signaling and plasticity mechanisms [35]. Furthermore, this calcium influx activates calcium/calmodulin-dependent protein kinase II ‎‎(CaMKII), a multifunctional enzyme abundant in glutamatergic synapses, significantly contributing ‎to calcium signal transduction, synaptic function, and memory-related processes [36]**‎**. This modulation is critical for sustaining the synaptic alterations that are crucial for underpinning memory retention.

### Nitric oxide regulation of synaptic proteins

Nitric oxide can influence the activity of protein kinases and phosphatases to regulate the phosphorylation status of important proteins and transcriptional factors, importantly CREB (cAMP response element-binding protein), which regulates genes associated with synaptic development and plasticity [37]. To identify the role of CREB in memory consolidation, scientists studied mutated mice with genetic loss of CREB function, in which the formation of LTM was affected without disturbing STM [1]. CREB functions by targeting the transcription of neuronally important genes, including c-fos, activity-regulated cytoskeleton-associated protein (Arc), and brain-derived neurotrophic factors (BDNF). It has been suggested that CREB regulates STM via the activation of above-listed genes, such as BDNF [38, 39]. The Morris water maze, a hippocampal-dependent spatial memory task, has shown that nitric oxide (NO) signaling deficiencies impair long-term potentiation (LTP) and reduce CREB activation via protein kinase A alterations, resulting in profound deficits in the consolidation of long-term spatial memories, emphasizing the critical interplay between NO, CREB, and hippocampal synaptic plasticity in learning and memory [40].

### Nitric oxide impact on AMPA receptors

AMPA receptors (AMPARs) are glutamate-gated ion channels, and their main function is to mediate fast excitatory synaptic transmission [41]. Nitric oxide facilitates the insertion of AMPARs into the postsynaptic membrane, indirectly by activating the cGMP-dependent cascade [42]. The NO-cGMP-PKG (cyclic guanosine monophosphate-protein kinase G) signaling pathway possibly promotes synaptic plasticity and fear memory formation by activating the ERK/MAPK signaling cascade [43]. NO activates soluble guanylate cyclase (sGC), increasing cGMP levels, and activating PKG. The activation of PKG facilitates the incorporation of AMPAR postsynaptically, thus increasing the number of receptors available for synaptic transmission to expedite the LTP [42, 43]. Pavlovian fear conditioning is conducted via This test involves pairing a neutral stimulus with an aversive stimulus to establish an associative memory, where this test shows that Reduced NO signaling has been shown to impair memory retrieval and synaptic plasticity, including AMPA receptor-mediated activity, in such paradigms [44].

### Crosstalk between NO and other gasotransmitters in memory processes

CO (carbon monoxide), H_2_S (hydrogen disulfide), and NO (nitric oxide) are endogenous signaling molecules called gasotransmitters that transmit chemical signals, which induce various physiological and biochemical changes. CO signaling is a molecular regulator of the NO feedback ring that can in turn stimulate CO synthesis by modulating the level of HO-1 protein, which in turn, can influence the formation of NO [45]. Both NO and CO play a role in synaptic plasticity and memory formation by activating the soluble guanylate cyclase (sGC) [46]. H2S also overlaps with NO signaling, complementing NO bioavailability by stabilizing nitrosothiol or reacting with reactive oxygen species (ROS) [46], thereby exerting its protective role as anti-apoptosis [45], which is crucial for regulating memory-related signaling pathways. Playing together, these Gasotransmitters engage in performing crucial role in memory and other neurophysiological processes by regulating vascular tone, inducing neuroprotection, and consolidating synaptic plasticity [46].

### Experimental evidence supporting NO’s role in memory consolidation

Multiple studies have been conducted to provide supporting evidence that nitric oxide does have a role in memory consolidation. A pharmacological inhibitor of NO was used to evaluate whether NO-mediated LTP has an essential role in memory consolidation or not. The NOS inhibitors that have been assessed are N-nitro-L-arginine and NG-methyl-L-arginine and were used intracellularly and extracellularly. The obtained results revealed that NO is a crucial contributor to enhancing the hippocampal LTP by reinforcing the involved synapses and facilitating the memory circuits [47]. Another study evaluated the involvement of PKG in LTP by using specific inhibitors and activators to target this kinase. This study showed that PKG inhibitor blocked the LTP induction while the use of PKG activator produced activity-dependent long-lasting enhancement suggesting that guanylyl cyclase and PKG are potent contributors to support the LTP. The activation of these molecules is tightly dependent on NO availability [48]. Another study used the Morris-water maze (MWM) in mice lacking nNOS (knocked-out) to study the learning and memory functions and cognitive abilities. Most of the nNOS knocked-out mice failed to find the submerged platform within the given time indicating that these mice had impaired spatial performance due to the direct effects of nNOS dysfunction [49].

### NO-Mediated epigenetic modifications in memory consolidation

NO plays a vital role in regulating epigenetic modifications, which are vital for memory consolidation. One mechanism is facilitating the dissociation of histone deacetylase 2 (HDAC2) from CREB-regulated gene promoters, via the activation of histone acetylation [50]. HDAC inhibitors can be used to increase histone H3 acetylation, by setting back the deficit observed in nitric oxide synthase (NOS) knockout mice [51]. Histone acetylation can overlap with the DNA methylation pathway. This is observed in impaired DNA methylation in the hippocampus due to NMDA receptor antagonists proposing a possible involvement of NO-relating signaling pathways in coordinating these epigenetic processes​ [52]. Additionally, the NO can affect chromatin remodeling in neurons and S-nitrosylation of HDAC2, which are essential for activity-dependent gene regulation during neural development and learning [52].

### Nitric oxide dysregulation and memory-related disorders

Nitric oxide has a dual role of being neuroprotective at physiological levels and neurotoxic when being overproduced [53]. Thus, there is no doubt that any dysregulation in nitric oxide levels would result in severe neurodegenerative and psychiatric disorders. Alzheimer’s disease (AD) is a distinct example of a neurodegenerative disorder where changes in NO signaling contribute to synaptic dysfunction and cognitive impairment [54]. Parkinson’s disease (PD) is another neurodegenerative condition that also exhibits malfunctioning NO signaling which causes defective motor and cognitive functions [55]. Additionally, psychiatric conditions, including schizophrenia [56] and depression [57] have been associated with abnormalities in NO pathways, emphasizing the molecule’s importance in brain function and memory.

In recent years, drugs targeting NO pathways have gained popularity. For example, selective iNOS inhibitors like 1400 W at 15 mg/kg per day for 2 weeks are being studied in preclinical trials for their potential to reduce neuroinflammation in Alzheimer’s disease [58]. Similarly, NO donors such as glyceryl trinitrate and cGMP pathway modulators such as sildenafil have shown a potential for improving memory and cognitive performance without altering amyloid burden in animal models and preliminary clinical trials [59]. Antioxidants such as N-acetylcysteine have been also investigated for their capacity to prevent nitrosative stress and the related cognitive impairment manifested in psychiatric disorders such as bipolar and major depression [60].

### Alzheimer’s disease (AD)

Alzheimer’s disease (AD) dementia is defined as a particular onset and progression of age-related cognitive and functional decline with a specific neuropathology [61]. The dysregulation of nitric oxide associated with advanced age and vascular dysfunction are contributing risk factors for AD [62]. To have proper cognitive function, nitric oxide is needed for neuronal communication and synaptic plasticity. In AD, the dysregulation of nitric oxide leads to multiple pathological features such as exacerbation of amyloid beta (Aβ) plaques deposition, disruption of synaptic signaling and plasticity, and chronic Inflammation. The overproduction of Aβ plaques exacerbates when nitric oxide is dysregulated causing the upregulation in the level of reactive oxygen species (ROS), particularly peroxynitrite [63, 64]. The overproduction of ROS accelerates cellular damage and leads to synaptic dysfunction [65]. Microglia produce neurotoxic NO [66] when they are activated during an inflammatory response to extracellular Aβ plaque deposition in the brain. Increased quantities of Aβ plaques can directly attach to mitochondrial outer and inner membranes changing their dynamics and functions and causing an aberrant energy metabolism which ensued a synaptic function loss [65]. A chronic inflammation is accompanied with a sustained high level of induced NO (iNOS) resulting and abnormal level of NO which further contribute to the deposition of Aβ plaques [67]. Overproduction of NO can also aggravate the synaptic plasticity disruption by altering the NMDA receptors expression, in which excessive NMDA receptors activation would cause neurotoxicity and promote cell death [68]. This can cause synaptic loss and contribute to the memory deficit observed in AD. Collectively, these mechanisms highlight the importance of restoring nitric oxide homeostasis as a possible prevention strategy or curing approach for Alzheimer’s.

### Parkinson’s disease

Defined as a chronic neurodegenerative disease mainly of the central nervous system that affects both motor and non-motor symptoms, such as tremors, rigidity, bradykinesia, cognitive deficits, and memory impairment. Initial studies have proposed that the dysregulation of NO signaling may play a role in the cognitive deficit in PD. Since NO plays a pivotal role in synaptic plasticity and the maintenance of LTP, these studies suggested that the dysregulation of NOS enzymes in PD patients might result in nitric oxide dysregulation which triggered a noticeable deficit in both the spatial and declarative memories that commodification observed in PD patients [69]. Correspondingly, postpartum studies performed in Parkinson’s patients’ brains and 1- methyl-4-phenyl-1,2,3,6- tetrahydropyridine MPTP-treated mice revealed that nitric oxide plays a significant part in PD, where MPTP-induced nigral dopaminergic neuronal death in Parkinson’s patients. Glial cells convert MPTP to MPP + to be utilized by dopaminergic neurons through dopamine transporters. Accumulation of MPP + hinders complex I of the mitochondrial electron transport chain causing DNA damage and activating PARP-1, ultimately leading to cell death. Numerous studies indicated that NO has a role in MPP+-induced DNA damage, PARP-1 activation, and cell death [70]. Similarly to AD, the formation of peroxynitrite can contribute to neuronal death and cognitive decline in the dopaminergic system and other brain regions involved in memory processing such as the hippocampus and cortex, in addition to inflammation that would activate the microglial, leading to upregulation of iNOS [71], hence exacerbating the memory deficit. Thus, therapeutic targeting of NO offers a possible strategy to alleviate cognitive decline in PD. Inhibiting nNOS and iNOS and applying antioxidants were demonstrated to relieve the effects of nitrosative stress in PD [72], suggesting that restoring NO homeostasis could improve cognitive outcomes.

### Schizophrenia

Schizophrenia is characterized by having psychotic symptoms, social and occupational decline with concomitant cognitive impairment [73]. Studies indicated that individuals with schizophrenia exhibited a significant disturbance in NO levels in the brain structures (cerebellum, hypothalamus, hippocampus, striatum) and fluids [56]. Several mechanisms of NO dysregulation possibly contribute to memory deficit in schizophrenic patients including impaired synaptic plasticity, altered glutamate signaling, and oxidative stress. Impaired synaptic plasticity in schizophrenic patients might be attributed to mitochondrial dysfunction leading to multiple manifestations, including altered ROS level and nitric oxide production [74]. This dysregulation in NO can disturb the LTP, causing memory formation impairment, as well as the excess ROS would damage brain regions like the prefrontal cortex and hippocampus, which are vital for memory. NMDA dysfunction seems to be particularly relevant in schizophrenic patients which have been validated by using NMDA antagonists that produced symptoms resembling schizophrenia manifestations [75]. Dopamine is believed to play a central role in schizophrenia as its increase contributes to the positive symptoms of schizophrenia, while its decrease contributes to the negative symptoms. Dopamine was also linked to the process of memory encoding and consolidation, particularly by stimulating the dopamine receptors. Stimulation of dopamine receptors as part of a hippocampal–striatal–prefrontal loop orchestrates the formation of new memories [76]. Thus, dysregulation of dopamine due to nitric oxide dysfunction would result in producing various symptoms related to schizophrenia. In schizophrenia, the dysregulation of NO triggers a series of molecular and cellular cascades that contribute to memory impairments. Maintaining balanced NO signaling is crucial for reviving the cognitive function in schizophrenic patients and can be purported as a potential therapeutic target for schizophrenia.‎.

### Depression

Nitric oxide is known for its influence on mental health in several pathways. Nitric oxide has a vital role in the pathogenesis of major depressive disorder by modulating the level of norepinephrine, serotonin, dopamine, and glutamate. These major neurotransmitters are involved in the neurobiology of major depression [77] and their disturbance is linked mechanistically with memory dysfunction in depression. A clinical study confirmed that the production of NO is increased in depressed patients [78]. NO influences memory deficit in depression as it plays a role in impairing the process of neurogenesis by instigating a disturbance in the level of NMDA receptors and neurotransmitters and enkindling baneful levels of oxidative stress and neuroinflammation. Accumulated reports have indicated that depression may be linked to the lack of hippocampal neurogenesis, which is negatively influenced by neuronal nNOS-derived NO, thus suggesting that the overexpression of nNOS in the hippocampus is necessary for chronic stress-induced depression, and inhibiting nNOS signaling in the brain may mitigate these debilitating effects [79].‎ Moreover, the dysregulation of NO signaling can hinder the regenerative abilities of neural stem cells (NSCs) causing an exacerbation in cognitive impairment associated with depression. As mentioned before, NO modulation of NMDA receptors also contributes to the memory deficit observed in depression. A study exposed that the antidepressant-like effect of the yueju pill on mice through the inhibition of the NMDA/NO/cGMP pathway providesREF12 evidence that supported the importance of NMDA receptors in memory deficit found in depressed patients [80]. NO modulates the neurotransmitter levels of both serotonin and dopamine which are implicated in mood regulation. Tetrahydrobiopterin (BH4) is a vital enzymatic cofactor required for the synthesis of serotonin, dopamine, and NO; thus, its dysregulation has been documented in many pathological situations, including Alzheimer’s disease, Parkinson’s disease, and depression as it increased the oxidative stress and neuroinflammation which can worsen the memory deficit exhibited in depressed patients [81]. The dysregulation of NO through its interaction with the serotonin transporter (SERT) negatively affects serotonin uptake by reducing SERT’s cell surface localization, thereby causing further deterioration in the depressed symptoms due to low availability of serotonin [82].

### Bipolar disorder

Nitric oxide is crucial for memory consolidation as it promotes LTP and retrograde communication in the hippocampus and prefrontal cortex [83]. Neuronal nitric oxide synthase (nNOS) regulates NO production, which modulates synaptic strength and creates lasting memory circuits [84]. ‎ In bipolar disorder, the NO signaling pathway is profoundly affected. A case-control study showed that bipolar disorder patients with mania had lower nitric oxide (NO) levels than healthy controls, but the difference was not statistically significant. The study found a modest negative association between NO levels and disease duration, which could have consequences for the neurobiology of bipolar disorder during manic episodes [85]. Abnormal NO production, either excessive or insufficient, can cause nitrosative stress and impede neuroplasticity [86]. Investigating the molecular imbalances in NO homeostasis may act as a link between neuroinflammatory processes and cognitive dysfunction in bipolar disorder, potentially leading to targeted therapeutic interventions.‎.

### PTSD

Nitric oxide (NO), predominantly through neuronal nitric oxide synthase (nNOS), influences synaptic plasticity by altering neurotransmitter release and LTD, as both are necessary for memory and learning processes. In PTSD, abnormal NO signaling, including excessive NO production and NO-induced post-translational modifications such as S-nitrosylation, impair these processes, contributing to defective neuroplasticity and the persistence of maladaptive fear responses [87]. Elevated stress levels related to PTSD cause dysregulation of the hypothalamic-pituitary-adrenal (HPA) axis, leading to altered glucocorticoid secretion, which can modulate stress-related physiological responses [88]. Neuronal nitric oxide synthase (nNOS) activation by calcium influx via NMDAR, is critical for the fear learning stages related to PTSD. Pharmacological interventions targeting nNOS have been demonstrated to limit fear acquisition and fear memory while improving extinction consolidation, making these drugs a prospective treatment options for PTSD patients [89].

## Conclusion

**Fig. 3:**
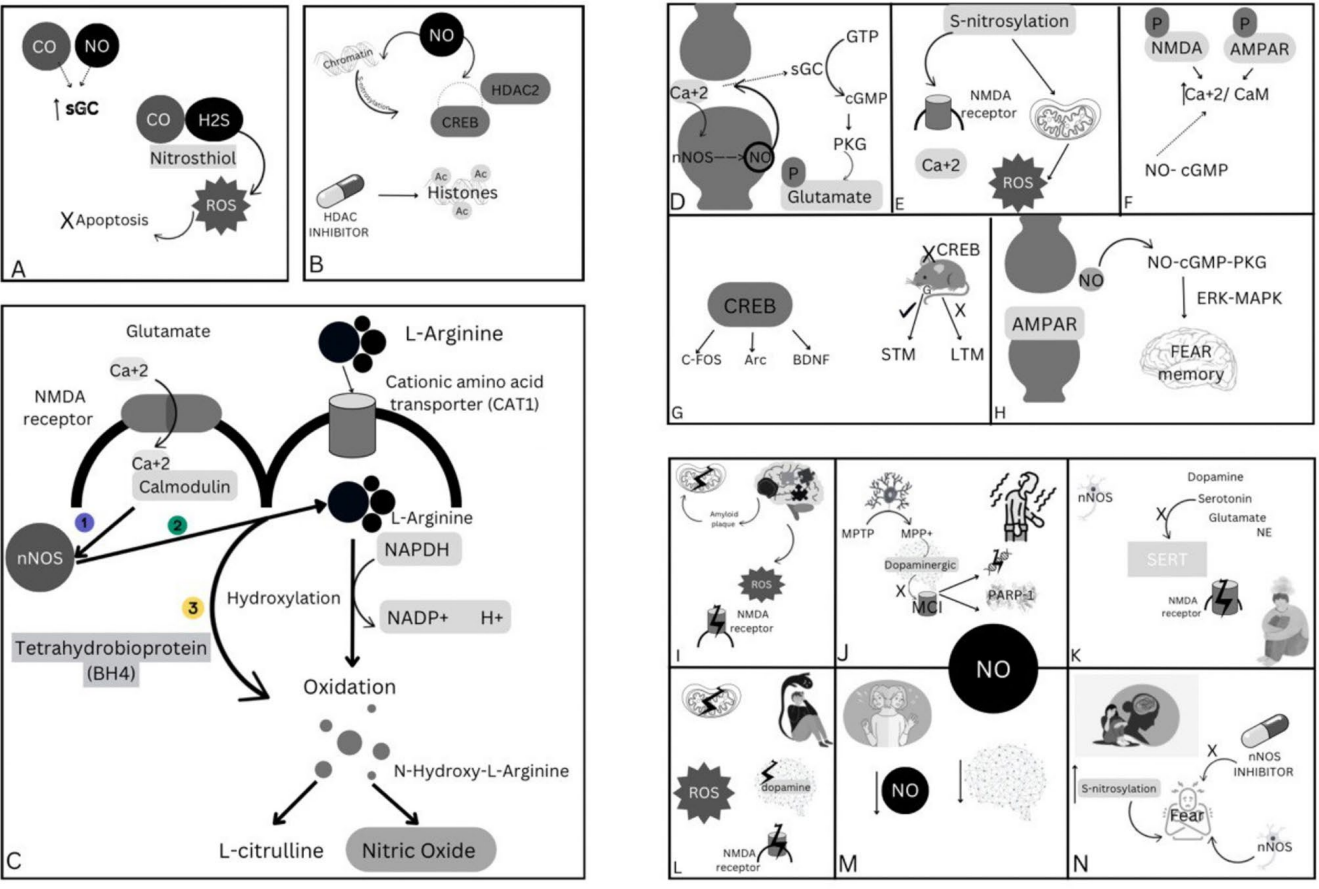
Demonstrates a full graphical summary. (**A**) shows the Cross-talk Between NO, with CO and H_2_S, (**B**) shows NO-Mediated Epigenetic Modifications in Memory Consolidation, (**C**) The biochemical steps for NO synthesis and the important molecular factors required for its production, (**D**) nitric oxide retrograde messenger, (**E**) nitric oxide modulation of s-nitrosylation, (**F**) nitric oxide modulation of CaMKs, (**G**) nitric oxide regulation of synaptic proteins, (**H**) nitric oxide impact on AMPAR, (**I**) nitric oxide role in Alzheimer’s, (**J**) nitric oxide role in Parkinson’s, **K**. nitric oxide role in depression, **L**. nitric oxide role in schizophrenia, **M**. nitric oxide role in bipolar disorder, **N**. nitric oxide role in PTSD

NO plays a pivotal role in memory consolidation through different molecular pathways such as acting as a retrograde messenger, modulating neurotransmitter release, regulating synaptic proteins, and influencing gene expression and protein synthesis. Thus, its dysregulation can negatively impact the synaptic plasticity in multiple neurological diseases, such as Alzheimer’s disease, Parkinson’s disease, schizophrenia, depression, bipolar, and PTSD, making it a potential therapeutic target. A schematic summary has been demonstrated in Fig. 3.

### Challenges and future directions

Our review highlights several critical gaps in the current understanding of the precise molecular pathway involving NO dysregulation and memory-related disorders. Future research might employ single-cell RNA sequencing to map NO-related gene expression changes during memory consolidation in specific regions of the brain. Furthermore, CRISPR-based genome editing could help identifying the significance of certain nNOS-associated genes in synaptic plasticity. Future studies to monitor NO metabolites in cerebrospinal fluid could aid in the identification of prognostic biomarkers for neurological disorders. Addressing these issues can help in bridging the current gaps related to the role of NO in memory and cognition and enhance the scientific understanding and clinical management of memory-related disorders involved nitric oxide dysregulation and to bring Moreover, advanced imaging techniques should be explored to study the spatial and temporal dynamics of NO in memory consolidation, further advancing this field of research.

## Data Availability

No datasets were generated or analysed during the current study.

## Abbreviations

NO: Nitric oxide
STM: Short-term memory
LTM: Long-term memory
NOS: Nitric oxide synthase
CAT-1: The cationic amino acid transporter
eNOS: Endothelial NOS
nNOS: Neuron NOS
iNOS: Inflammatory NOS
BH4: Tetrahydrobiopterin
FAD: Flavin Adenine Dinucleotide
FMN: Flavin Mononucleotide
LTP: Long term potentiation
NMDA: N-methyl-D-aspartate
sGC: Soluble guanylate cyclase
CaMK: Calcium calmodulin-dependent kinase
Arc: Cytoskeleton-associated protein
BDNF: Brain-derived neurotrophic factor
NO-cGMP-PKG: Cyclic guanosine monophosphate-protein kinase G
MWM: Morris-water maze
AD: Alzheimer’s disease
PD: Parkinson’s disease
